# How Could Agroecological Markets Support Healthier Diets in Urban and Periurban Areas for Low-Income Women? A Case Study of Dakar, Senegal

**DOI:** 10.1016/j.cdnut.2026.107719

**Published:** 2026-05-20

**Authors:** Rachel Bezner Kerr, Moustapha Seye, Arlène Alpha, Ninon Sirdey

**Affiliations:** 1 Ashley School of Global Development and Environment, Cornell University, Ithaca, NY, United States; 2 Research Département des Sciences Humaines de l'Institut fondamental d'Afrique noire in the Université Cheikh Anta Diop De Dakar (UCAD), Dakar, Senegal; 3 CIRAD, UMR MoISA, Montpellier, France; 4 MoISA, Univ Montpellier, CIRAD, CIHEAM-IAMM, INRAE, Institut Agro, IRD, Montpellier, France

**Keywords:** agroecology, malnutrition, West Africa, urban, diet-related noncommunicable disease

## Abstract

**Background:**

Agroecology is a holistic approach that draws on ecological and social justice principles. Although there is evidence that agroecological practices can improve food security and nutrition for rural households, there is less research in urban areas.

**Objectives:**

This exploratory study in Dakar, Senegal, asks: how could agroecological markets support healthier diets in urban and periurban areas for low-income consumers?

**Methods:**

Four market initiatives were studied that were urban/periurban, sold agroecological food products, and had low-income consumers. Three focus groups were held with women consumers, and interviews were conducted with consumers and those involved in agroecological markets. A survey was carried out with 180 consumers in 6 market sites.

**Results:**

Small-scale agroecological market initiatives were well established, relied on short chains built on trust that promoted values such as health, reduced use of chemicals, and fairness. Women who purchased agroecological foods regularly bought a diverse range of foods. One-third reported having a diet-related disease such as diabetes. Women reported consuming agroecological products either as prevention or after being diagnosed with a diet-related disease. Main motivations for consumers to purchase agroecological foods were health, taste, and avoidance of agrochemicals. Consumers noted several barriers to eating more agroecological foods, including market availability, distance, and seasonal availability of food products. Seven pathways to connect agroecology to nutrition were found: *1*) agrobiodiversity, *2*) livelihoods/social empowerment, *3*) knowledge cocreation, *4*) participation/connectivity, *5*) culture and diets, *6*) reduced exposure to pesticides, and *7*) rights-based approaches.

**Conclusions:**

Six recommendations were identified to promote these pathways, including support for agroecological producers in urban low-income areas, support for short agroecological markets, and strengthening urban food governance. Although not generalizable, the conceptual framework addresses research gaps on the agroecology–nutrition nexus by providing food for thought for the design of interventions that aim to support agroecology and nutrition of urban low-income consumers.

## Introduction

Food systems are not yet delivering nutrition, climate resilience, or other sustainable development goals. Undernutrition and micronutrient deficiencies persist alongside rising rates of obesity and diet-related noncommunicable diseases (NCDs) [1]. Recent publications on the nexus of planetary boundaries, justice, nutrition, and health showed that more diversified food systems are needed to deliver positive health, social, and environmental outcomes [[2], [3], [4]]. In Africa, rapid urbanization, changing diets, high income inequality, and high vulnerability to climate change impacts [1,5] mean that agri-food systems must transform to ensure equitable access to healthy, nutritious, and sustainably produced food for low-income households [6,7]. Agroecology is a holistic approach to food systems that draws on ecological and social justice principles, with evidence that such an approach serves as a pathway to improved nutrition [[8], [9], [10]]. Most studies, however, are drawn from empirical data in rural contexts, with few studies focusing on urban consumers’ nutrition, particularly overnutrition and diet-related NCDs such as diabetes. Deaconu et al. [11] compared the diets of agroecological and nonagroecological farmers by assessing consumption of processed foods, BMI (in kg/m^2^), and self-reported diagnosis of diet-related diseases. They found that agroecological farmers had healthier dietary patterns (although equally high prevalence of overweight/obesity), but they did not consider consumers in urban areas.

Lastly, most of the studies tend to focus on agroecological *practices* at the farm or field scale (e.g., crop diversification, agroforestry), rather than higher levels of agroecological transitions. Other dimensions of agroecology, such as fair markets, governance, and direct producer–consumer relationships, are far less studied. Existing studies on agroecological markets, sometimes called “nested” or “peasant” markets, have been mostly in North America, Europe, Latin America, or Asia [8,12,13]. In Ecuador, for example, April-Lalonde et al. [14] conducted a study in 3 cities on the motivations of consumers who purchase their food from agroecological markets and found that personal health was a major motivation and that their diets were higher in fruits and vegetables and lower in processed foods high in salt. Africa is particularly understudied, with only an emerging literature, for example, on participatory guarantee system (PGS) efforts in Morocco [15] or on the diversity of local organic markets in Kenya [16].

Bezner Kerr et al. [8,10] suggest that research on agroecology–nutrition linkages should consider urban areas, NCDs, and socioeconomic dimensions of agroecology when adopting a food system approach. Furthermore, a critical challenge for agroecological food systems is to improve the nutrition of low-income urban consumers. A crosscutting analysis of 30 programs related to agroecology in 6 West African countries showed that consumers of certified agroecological products are often from upper-income households [17]. The present study explores how agroecological food systems could contribute to nutrition, with a focus on women of reproductive age living in low-income urban areas, and on fresh fruits and vegetables. We aim to address the research question “How could agroecological markets support healthier diets in urban and periurban areas for low-income women?” with 3 subresearch questions: *1*) How do the main marketing circuits of locally produced agroecological fruits and vegetables work? *2*) What are the factors influencing the supply chains for agroecological markets, consumer behaviors, and diet? *3*) What are the potential impact pathways from agroecological markets to safer and more diverse urban and periurban diets for low-income women? By addressing these questions, we aim to go beyond agroecological production practices to consider other dimensions of agroecology, such as food governance, social inequities, and connectivity between farmers and consumers [18,19].

The study was implemented in Senegal, a West African country that illustrates the triple burden of malnutrition, with high rates of child stunting (17.9%), although much lower than the mean for the Africa region (30.7%) [1], and higher anemia rates among women aged 15 to 49 y (52.7%) compared with the Western Africa average (48%) in 2019 [20]. Concurrently, the prevalence of overweight and obesity has increased sharply over the last 2 decades and now affects 29% of women aged 15 to 49 y, with women living in the region of Dakar having the highest prevalence (41.1%) [21]. Similarly, low-income women are particularly exposed to overweight/obesity [22]. These multiple forms of malnutrition generate diet-related NCDs and are linked to poor-quality diets. A food consumption analysis carried out in Senegal confirms that energy-dense, sugary, salty, and fatty foods are consumed between 6 and 7 d a week, whereas vegetables are consumed around 5 d a week, and fruits 4 d a week [23]. In addition, the wealthier and more educated households consume more fruits and vegetables [24,25], whereas poorer neighborhoods exhibit a more frequent supply of highly processed foods in their food environment than better-off areas [26].

Senegal also has an active social movement on agroecology, “*Dynamique pour une Transition Agroécologique au Sénégal*” (DyTAES), which, since 2019, has brought together nongovernmental organizations (NGOs), farmer unions, research institutions, government actors, donors, and the private sector, and has gained legitimacy and effectiveness in advocating for agroecology [[27], [28], [29]]. A presidential decision in 2019 made agroecology one of the government’s priorities [29,30], and 10% of the budget allocated to fertilizer subsidies was devoted to organic fertilizers in 2021. Over the past decade, many projects supporting agroecology have been implemented, led by farmers, civil society organizations, international agencies, or governmental and intergovernmental institutions [[31], [32], [33], [34]]. Few of them, however, aim to promote agroecological markets or to reconnect consumers and producers through alternative food networks (AFNs) [28,29]. By exploring the agroecological approach in a way that benefits both family farmers and urban consumers, especially the most vulnerable, the present study aims to contribute to the broader definition of agroecology [35] reflected by DyTAES [34]. Concretely, agroecology refers to a bundle of ecosystem-based practices with reduced chemical inputs or without them, intentionally implemented in the food system to protect agroecosystems and ensure quality food and connectivity, commonly understood in Wolof as “mbey mu sell,” that is, healthy agriculture.

## Methods

### Analytical framework

**FIGURE 1:**
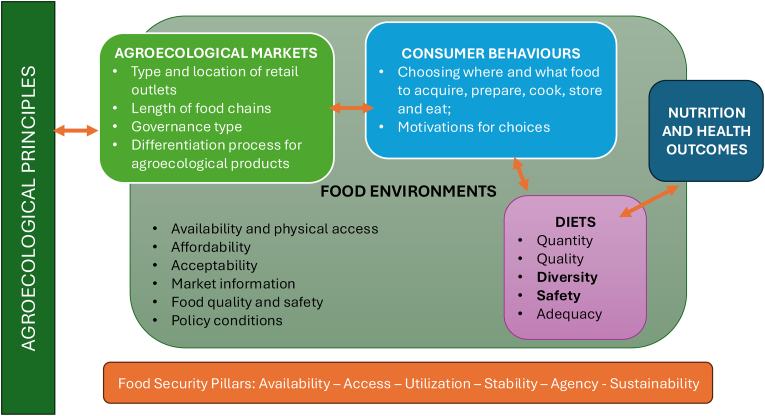
Analytical framework used in the paper (adapted with permission from: HLPE 2020, Sustainable Food System Framework, Figure 2). HLPE, High Level Panel of Experts.

To analyze the potential for agroecological markets to support healthier diets among low-income urban households, we draw on recent work by the United Nations High Level Panel of Experts (HLPE), which highlights the significance of the food environment for shaping markets, consumer behaviors, and diets, and ultimately food security and nutrition outcomes [36]. Our analytical framework combines the 13 agroecological principles identified by the HLPE [35] with types of markets, consumer behavior, diets, and the food environment (Figure 1). We consider each of the 6 dimensions of the food environment, as shown in the HLPE framework [36], adapted to understand the factors that could influence the supply chains for agroecological markets, consumer behaviors and diets, and broader structural factors that shape the food environment of urban Senegal. Specifically, we consider the following 6 dimensions: availability and physical access, affordability, acceptability, market information, food quality and safety, and policy conditions (see Figure 1).

In addition, to characterize agroecological markets and their potential for improving nutrition, we developed an analytical grid by drawing on the literature related to AFNs, nutrition-sensitive value chains [37], and markets for agroecology [38]. This analytical grid characterizes agroecological markets in relation to: *1*) the type and location of retail outlets [37,39]; *2*) the length of food chains in terms of distance and number of intermediaries (direct sales or short distribution channels) [14,38,[40], [41], [42]]; *3*) the type of governance, which can be captive (when farmers are highly dependent on a client), market (when actors easily and frequently switch to new partners), or relational (when actors are independent but closely related through spatial and relational proximity, acquittance, and regular exchanges) [43]; *4*) the differentiation process (qualification, guarantee, and signal of quality), which includes self- or no certification (trust-based relationships), third-party certification, or PGS.

Our focus for diets was on dietary diversity and the frequency of fruits and vegetables being consumed as intermediate outcomes of nutritional status. The diversity of foods being consumed by individuals has long been recognized as an important characteristic of nutrient adequacy, a component of a healthy diet [44], and a relevant outcome of nutrition-sensitive agricultural interventions [45]. Scoping and systematic reviews suggest that there is a wide range of dietary diversity indicators and no clear, significant relationship between dietary diversity and health outcomes such as NCDs [44,[46], [47], [48]]. Instead, diet quality, in which assessment of dietary intake of nutrient-dense foods, including fruits and vegetables, and conversely reduced intake of sweets, sugar-sweetened beverages, and red meats, is considered a more reliable measure with health outcomes connected to diet-related NCDs [48]. We included consumption of fruits and vegetables as one indicator of diet quality in this exploratory study.

### Data collection

We used a mixed-methodology case study design combining qualitative and quantitative tools. Data collection was organized into 2 main components: *1*) a “market component” to document the functioning and the challenges of existing agroecological market initiatives in urban and periurban areas of Dakar; *2*) a “consumer component” to characterize the socioeconomic and dietary profiles of women consumers of agroecological products, to understand their motivations, and to identify their constraints in accessing agroecological products.

The research protocol was approved by the Institutional Review Board for Human Participant Research of Cornell University, and by the National Ethical Committee for Research in Health (Comité National d’Ethique pour la Recherche en Santé). The objectives of the study, as well as the rights of the respondents and the duties of the research team in terms of data protection, were explained to each respondent, and a consent form was signed by each. At the end of the interview, they were given an information sheet with project information and the research team’s contacts.

### Market component

On the basis of exploratory fieldwork of 2 of the authors to meet various public and private actors involved in agroecology, as well as the long in-country experience and the personal network of 2 of the authors, 12 existing agroecological market initiatives were identified in urban and periurban areas (Table 1). We used the market analytical grid to screen and characterize each of them. The 2 main types of marketing channels are direct sales from farmers to consumers (5 initiatives) and short chains with 1 intermediary (6 initiatives), whereas 1 initiative is a long chain with 2 or more intermediaries. Only 1 initiative sells certified organic products based on a PGS called BioSenegal. Therefore, most of these initiatives can be considered as agroecological initiatives without any guarantee system.

**TABLE 1:** Synthesis of the inventory according to their location, type of channels, and likelihood to reach low-income urban and periurban consumers.

Market type and location	Periurban	Urban
**Direct sales**	Toubab Dialaw^3^/Ferme des 4 chemins^1^ Mampuya	Thiaroye Gare^1^ Patte d’Oie^1^^,^^3^ Passion Nature^2^ Amitié 2^1^
**Short distribution channels (1 intermediary)**	Comité d'initiatives pour la gouvernance alimentaire in Bambilor^1^^,^^3^ Calebasse Verte^2^	Sell Sellal (certified products by BioSenegal) Bertha market^2^ Thiès^1^^,^^3^
**Long distribution channels (2 intermediaries or more)**		Lendeng^1^ (transition toward agroecology)

1 Likely to reach low-income urban or periurban consumers.

2 Using online distribution.

3 Selected for in depth study.

We then used a case study research approach to select some of these initiatives for an in-depth study, which means that case studies were selected not for their representativeness but for their relevance in achieving both objectives of agroecological markets and benefiting urban low-income women. Four contrasted initiatives were selected in this perspective based on the following criteria: *1*) products are sold in urban/periurban areas; *2*) products are claimed to be agroecological or organic, and *3*) low-income consumers are some of the market clientele.

Semistructured interviews were conducted with the selected case studies, either in Wolof or in French, with farmers, vendors, and supporting actors (e.g., NGOs), who were identified during fieldwork and based on the snowball technique (*n* = 13). The interview guide addressed the following topics: *1*) the history, motivations, and objectives of the agroecological initiative; *2*) the concrete organization of marketing channels; *3*) product differentiation in the market; *4*) the interface with customers; and *5*) the challenges faced and any recommendations. All the interviews were transcribed and analyzed through a thematic analysis. A webinar was organized with most of the interviewed stakeholders to share and discuss preliminary findings.

### Consumer component

The methodology used for the consumption component included qualitative methods and quantitative surveys conducted in 6 locations. The 4 selected markets were extended to other similar sites identified based on the snowball method to reach the targeted sample: Patte d’Oie, Amitié 2, Bambilor, Toubab Dialow, Thiès, and Pout Diack. The qualitative methods included semistructured interviews, focus groups, and direct observations of 56 people in total, including interviews with female consumers of agroecological products (12), nonconsumers of agroecological products (17), resource persons (sellers, NGO staff, and local authorities) (10), and focus groups with 17 participants who were female consumers of agroecological products (Table 2).

**TABLE 2:** Breakdown of qualitative sample size according to point-of-sale origin and type of participation.

Types of participation	Focus groups	Consumer interviews	Nonconsumer interviews	Resource persons	Total interview
Urban: Patte d'Oie/Amitié 2	5	4	5	4	18
Urban/rural Thiès/Pout Diack	5	4	6	3	18
Periurban: Toubab Dialaw/Bambilor	7	4	6	3	20
Total	17	12	17	10	56

The quantitative survey was carried out with 180 women consumers of agroecological products of women aged 18 to 49 y (Table 3). The questionnaire covered the following topics: socioeconomic profiles, eating habits, consumption of agroecological products, health and medical history, motivations, (physical and economic) access to agroecological products, purchasing and consumption behavior, relations with producers/sellers, and prospects for the development of agroecology. In addition, BMI measurements were conducted by the enumerators for 141 women who knew their height (and/or had their ID documents with them) and by using a scale. For 39 women, it was not possible to measure their BMI because they did not know their height or did not have their identification documents, and because the scale used in Thiès broke during the survey. To assess the dietary diversity of women consumers, a 7-d recall was included in the eating habits section of the questionnaire. We applied the same 18 food groups as those used in the most recent national survey on food security, nutrition, and resilience [21]. These food groups were different from the ones required to measure the Minimum Dietary Diversity of Women of Reproductive Age. As an exploratory case study, our objective was primarily to characterize women’s diets and not to assess the degree of coverage of their nutritional needs.

**TABLE 3:** Breakdown of the quantitative sample according to origin by sales outlet.

Point of sale	*n*	Urban (%)	Periurban and rural (%)
Urban: Patte d'Oie/Amitié 2	60	93.3	6.7
Urban/rural: Thiès/Pout Diack	60	50.0	50.0
Periurban: Toubab Dialaw/Bambilor	60	90.0	10.0
Total	180	77.8	22.2

The distribution of the sample according to place of origin shows that the outlets in Patte d'Oie/Amitié 2 and Toubab Dialaw/Bambilor are mainly frequented by women from urban areas (93.3% and 90%, respectively), whereas Thiès/Pout Diack has a balanced distribution between urban and periurban/rural areas (50% each). Overall, 77.8% of survey respondents live in urban areas and 22.2% in periurban and rural areas.

### Limitations of the study

The study has some limitations that are linked to the project length, size, and the relatively few agroecological market initiatives in urban or periurban areas in Senegal. The sample size is relatively small, and the selection method used does not allow for generalization of the results. Given time constraints (9 mo) and budget, it was impossible to build evidence of the impact of agroecological markets on women consumers’ nutrition using methodology such as randomized controlled trials or quasi-experimental protocols. It was also not possible to have a direct assessment of diet-related NCDs through blood analyses, and we had to rely on self-reported data for diseases and for the height used for the BMI. This study is an exploratory study that identifies *potential* impact pathways of agroecological market initiatives on nutrition in urban and periurban areas, and relevant policy recommendations that could build on these impact pathways to improve nutrition. The research design and the size of consumer samples in particular were tailored for this purpose and not for identifying causal links between the consumption of agroecological products and nutrition outcomes.

We focused on contrasting cases of agroecological markets in only 1 country with limited field work; thus, this study does not represent all the potential organizational innovations in agroecological marketing channels. Furthermore, there was difficulty reaching the targeted number of women consumers in some of the identified markets, which led us to enlarge the geographical scope of the study to 1 rural area (Pout Diak). Finally, the study was implemented in the low season of vegetable production in Senegal, limiting direct observations, and price records are not well documented in the specific case studies. Overall, the findings, including the proposed conceptual framework and policy recommendations, are to be used as food for thought for the design of interventions that aim to support agroecology and nutrition.

## Results

### The market for agroecology in urban and periurban areas in Senegal

The in-depth study of the functioning of the 4 selected markets shows that 2 of them are direct sales initiatives (Patte d’Oie and Toubab Dialaw/Ferme des 4 chemins), and the other 2 are short food chains (Thiès and Bambilor).

### Patte d’Oie

**FIGURE 2:**
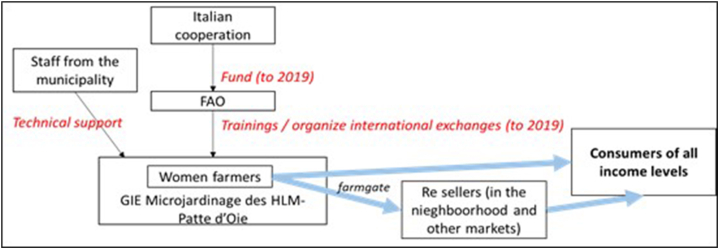
Diagram of Patte d'Oie initiative (Dakar). Actors are in boxes; product flows are represented in blue arrows; immaterial resources are represented in thin black arrows and red narratives. GIE, Groupement d'Intérêt Général.

Patte d’Oie (HLM Patte d’Oie is the name of one neighborhood of Dakar city) is located in the heart of Dakar and run by a women’s economic interest group called the “*GIE des femmes du micro jardinage d'HLM Patte d'Oie*.” These women were initially supported by Dakar city, the neighborhood committee, and the FAO. Since 2002, a series of programs have been run to train and support the women to produce spices, herbs, and vegetables on raised beds, tables, and pots (Figure 2). About 30 women are currently active, although there were 100 women at the start. The main products are mint and other herbs/spices (rosemary, aloe vera, basil, lemon balm, etc.), as well as vegetables such as beetroot, turnips, lettuce, and eggplants, with higher availability in the dry season and lower production in the rainy season. Most women focus on mint because it is a low-risk, high-income crop (mint can be harvested around 40 d after planting, and then every 5 d), unlike vegetables, which are vulnerable to attack from rats and whiteflies. Most vegetable production is self-consumed, and a very small portion is sold. The president of the organization estimated that they sold between 15 and 25 tables of mint each week, and between 60 and 90 pots of fresh herbs/spices per week. [This roughly represents 40 kg of mint and 40 kg of spices per week, according to her. The quantity varies widely along the year and even within each month (with more sales in the first part of the month compared with the end of the month).] They receive no support, apart from Dakar city’s staff who come on a regular basis to help them, for example, with the renovation of the tables.

The type and location of the retail outlet are favorable to a wide access of products because it is located in a low- to middle-income neighborhood, near a busy street. As a direct sale scheme, it favors direct exchanges between producers and consumers, including consumers, vendors, and street caterers (e.g., juice producers). They buy products through direct sale when they come to the garden, through prior orders, or by delivery (by motorbike), in small (herbs for 500 Communauté Financière Africaine, or CFA) or large quantities (e.g., a whole table). The organization of this market combines individual and collective management (Figure 2). Each woman works individually, building her own tables, managing her own supply of inputs [seeds, wood, substrate made from groundnut hulls, rice husks, laterite, and the mineral solution micro–macro (The macro is composed of MKP, calcium nitrate, and potassium nitrate. The micro is made up of magnesium nitrate, magnesium sulphate, copper sulphate, zinc sulphate, boric acid, ammonium molybdate, and sequestrene. Mamadou Sarr, « Approches didactiques de la problématique de l'eau en milieu formel et non formel au Sahel », *VertigO - la revue électronique en sciences de l'environnement* [En ligne], Hors-série 1 | décembre 2003, mis en ligne le 15 décembre 2003, consulté le 10 octobre 2024. URL: http://journals.openedition.org/vertigo/1964; DOI : https://doi.org/10.4000/vertigo.196)], and selling individually to their respective clients, with their own price-setting mechanism. In parallel, they manage collectively productive investments and some labor (such as renovating the water pump or paying young people from the neighborhood to do the watering), along with a collective savings scheme.

There is no formal differentiation process, nor qualification, guarantee system, or signal of quality. Their practices are in line with the microgardening training courses attended at the start of the project and are not described as “organic” but as agroecological, with no pesticides used and only a micromineral and macromineral solution used as a chemical input. This collective identity is not controlled and is shared with consumers by direct exchanges, with no sign at the entrance of the site.

### Toubab Dialaw

**FIGURE 3:**
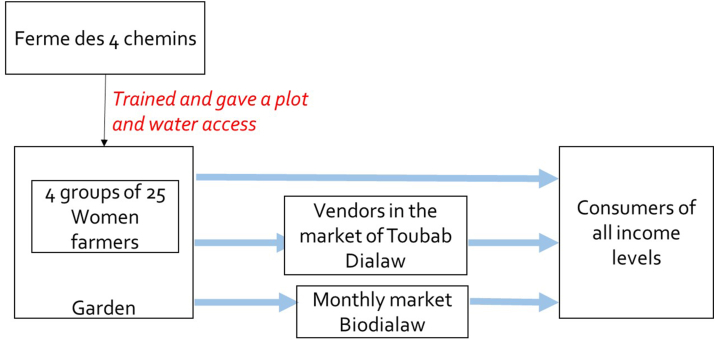
Diagram of the Toubab Dialaw initiative.

The Toubab Dialaw initiative is a 4-hectare demonstration diversified and integrated farm (e.g., market gardening, field crops, fruits, seed production, fish farming, beehives), which welcomes and trains a diversity of people. The leader of this demonstration farm supports 100 women organized in 4 groups for agroecological production and sales (Figure 3). They received 1 hectare of protected land with access to water and grow agroecological products. The philosophy is to embrace all agroecological principles to produce, trade, and consume healthy products with no chemical inputs and demonstrate that agroecology is viable. The 4 “Chemins” or pathways of the farm mentioned in the complete name of the initiative (*Ferme des 4 chemins*) refer to 4 critical dimensions of agroecology: education, environment, health, and sustainable development.

There are several locations of retail outlets. Beyond self-consumption, vegetable sales are made on-farm, through the marketing channels used by the farm (Whatsapp group and a monthly market organized on the demonstration farm “Marchés Biodialaw”) and through local sales at the Toubab Dialaw market (daily market). All these channels are direct sales. For the last channel, women buy vegetables from the collective at a lower price than their selling price and resell them individually at the market on their behalf. They set fixed prices according to production costs and not depending on seasonal production. This initiative, hence, also combines individual (sales) and collective management (production management, training, organic input, and water access). There are no formal specifications or guarantee system, but the products are signaled as agroecological during the monthly markets through the *Ferme des 4 chemins* signs. In the local market, no distinctive sign is used.

### Thiès

The organic market of Thiès (the third largest city of the country located 70 km away from Dakar) is held every Friday and Saturday for ∼20 y. Around 10 women vendors sell fresh fruits and vegetables, fresh herbs, dried herbs, processed local cereals, oils, and fish products. The women vendors sell between ∼150 and 200 kg of fruits and vegetables per week in the low season and 300 and 500 kg/wk in high season, which means between 12 and 20 tonnes per year. Around 10 organic vegetable farmers supply the market, with some women vendors also being farmers. Support for this market is led by the NGO Agrecol Afrique, part of a 20-y program to support food system actors. The NGO aims to bring synergies between all its activities to promote food sovereignty and healthy food systems.

**FIGURE 4:**
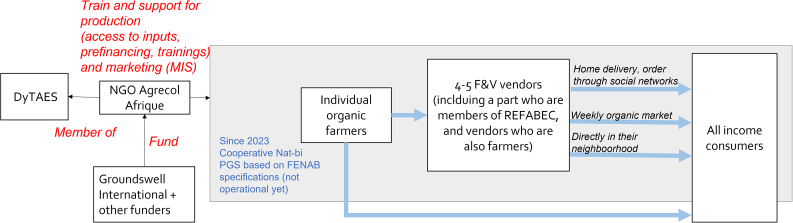
Diagram of the organic initiative in Thiès. DyTAES, Dynamique pour une Transition Agroécologique au Sénégal; FENAB, Fédération Nationale pour l’Agriculture Biologique; NGO, nongovernmental organization; PGS, participatory guarantee system; REFABED, Réseau des femmes en agriculture biologique et commerce équitable.

The organic market is frequented by consumers of all income levels, although it is not located within a low-income neighborhood. This is a short chain because market vendors buy their supplies directly from local organic farmers and sell to consumers. Relationships and transactions between farmers and vendors, and between vendors and consumers, are governed by trust, facilitated by relational and geographical proximity, and regular and direct exchanges of information. Agrecol Afrique also plays an intermediary role between actors, fostering trust, price-setting mechanisms, promotion, and access to inputs. To facilitate farmgate and retail prices setting, the NGO has set up a market information system, and recorded retail prices are shared between producers, sellers, and consumers. The NGO also prefinances farmers, supplying the inputs (seeds, organic fertilizer) to farmers who pay back the money at season’s end. The differentiation process is based on the specifications for organic farming in Senegal, drawn up by the *Fédération Nationale pour l’Agriculture Biologique* and used as a reference (Figure 4). Although a PGS has been constructed, it is not yet operational, and the guarantee of the quality is based on trust and word of mouth. The market does not use any market signs to communicate the quality of products sold, but they are located in a place far from the conventional market to stand out.

### Bambilor

**FIGURE 5:**
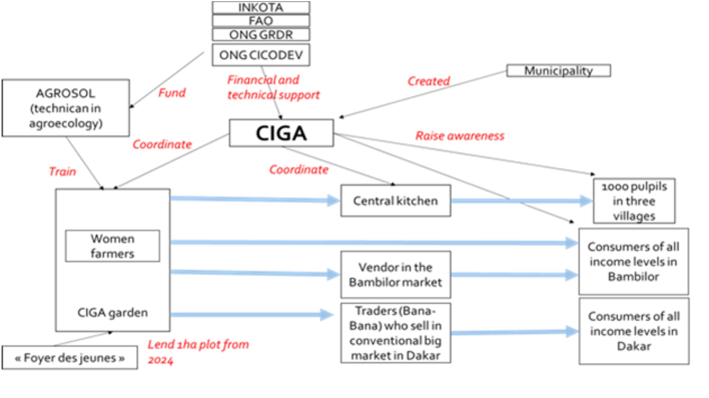
Diagram for the Bambilor initiative. CIGA, Comité d'initiatives pour la Gouvernance Alimentaire.

The Comité d'initiatives pour la gouvernance alimentaire (CIGA), set up by the Bambilor municipality (periurban Dakar region), has been implementing an agroecological garden initiative since 2021. The CIGA advocates for a holistic vision that consists of producing, marketing, preparing (in the central kitchen), and consuming agroecological products (through raising awareness of the importance of healthy diets for human health and nutrition). Bambilor is under land pressure. The initiative started with a small plot (2500 m^2^, of which 1000 m^2^ is under production) in 2022 with the support of FAO and 2 NGOs, Groupe de Recherche et de Réalisation pour le Développement Rural and Comité d'initiatives pour la Gouvernance Alimentaire (CICODEV) (Figure 5). Harvested vegetable volumes were low at the time of this research in 2024, but are expected to expand with the new plot of 1000 m^2^. The CIGA plans to pay 30% of the profits to young people who gave the land and will work in the garden as well, 40% to the women who work in the field, and 30% to the CIGA.

Although the main product is agroecological vegetables intended for a central kitchen supplying 4 school canteens (supported by the same projects), this production is supplemented by the local market, farm gate sales, and traders selling in a large conventional market in the center of Dakar. This market, therefore, combines direct and short-chain sales and various retail outlets. Governance mechanisms cannot be assessed so far because this was a small plot where very few people were working, all part of the CIGA. They stated that they produce vegetables without chemical inputs, but no formal specifications have been set, nor has a guarantee system or label/specific communication to consumers.

### Main characteristics and common features of the market initiatives

The analysis of the 4 case studies of agroecological market initiatives shows several common features. First, the location of retail outlets and length of channels are likely to favor access to agroecological products for low-income consumers because they are located in or close to low-income neighborhoods and they use direct or short channels, which are likely to keep transaction costs low and favor higher connectivity between producers and consumers. Second, transactions are governed by trust and reputation, with no formal specifications or guarantee system, which suggests relational governance. This approach is favored by spatial proximity and direct, regular exchanges between actors (both between vendors and consumers and between vendors and farmers). Yet, participatory certification is positively perceived. In Thiès, they are building their own PGS, and the farm visits included are seen as a way to further reinforce the trust between stakeholders (internally first, and then as a strength to attract more consumers). Third, collective action between peers exists in all the initiatives, mainly for access to services (e.g., training), inputs (e.g., organic fertilizers or seeds), land and infrastructure (e.g., market space, a water pump, farmland), as well as for ensuring solidarity. Market transactions, however, are individually run except in Bambilor, which enables flexibility in terms of quantity (small and big quantities), location (farmgate or garden, preorder and pick up, delivery), customers (loyal, new, households, organic or conventional resellers), timing (preorder by phone or direct), and payment (on delivery or by credit). In addition, in all the case studies, an intermediary actor (NGO or local authority) played a key role in the emergence and sustainability of the initiatives. Finally, although none of the cases use a label or sign to indicate the agroecological quality of their produce, they have a good reputation thanks to word of mouth and values shared by all stakeholders, from producers to consumers. They are based on healthy production practices (zero or reduced chemical inputs), local origin, and naturally based diets that are high in fruits, vegetables, and other nutrient-dense foods, and low in highly processed items. The intrinsic signs of quality reported by the actors were also the same: freshness, no chemicals, taste, and good storage capacity. Although they are small-scale initiatives, most of these initiatives (e.g., Patte d’Oie, Thiès, Toubab Dialaw) have existed for ∼20 y.

### Consumption of agroecological products in urban and periurban areas in Senegal

Women who frequented the various agroecological outlets were mostly married (73.3%), and women aged 35 to 45 were the most numerous (38.3%), followed by those aged 46 to 49 (30.6%) across all outlets. Younger age groups (18–24 and 25–34 y) represented a third of the total sample. Almost half (46.2%) of them had at least secondary education, whereas 32.2% had primary education, and they had overall higher than average levels of education compared with national rates.

### Employment status of consumer respondents

Overall, more than half of all women (53.3%) were in informal employment. The proportion of unemployed women was 22.8%, followed by those in regular formal employment (19.4%) and those in irregular formal employment (4.4%). Most unemployed women consumers in the areas studied were doing household work, with only a small proportion engaged in education or other unpaid activities. The consumers surveyed included a high proportion of self-employed women (71.2%) among those who were employed. Wage employment in the private (13.7%) and public (7.9%) sectors remains marginal, whereas agriculture accounted for 5% of declared occupations. These results confirm the importance of the informal and self-employed sector for women consumers, although the diversity of jobs was more marked in Thiès. Less than 1% of women worked in technical or formally structured fields such as industry or finance. Overall, these results suggest that most women who frequent agroecological markets have predominantly precarious or informal occupations, underlining persistent challenges of access to stable and structured employment for women in the study areas.

### Women consumers’ health and medical history

#### Women self-reporting NCDs

**FIGURE 6:**
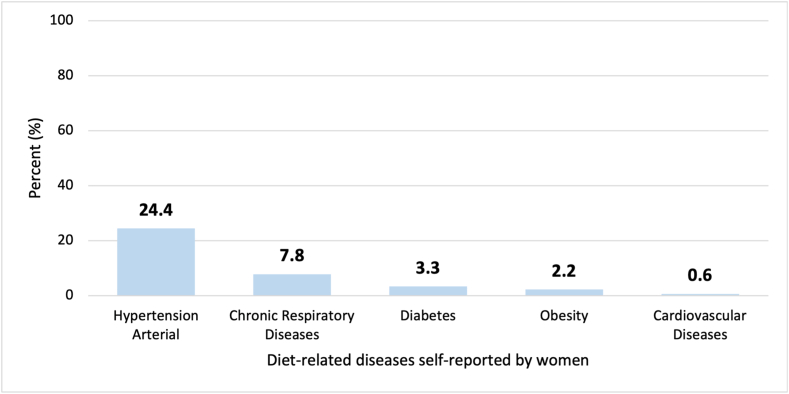
Proportion of diet-related NCDs self-reported by women (%) (*n* = 180 respondents). NCD, noncommunicable disease.

Women self-reported in the survey whether they had any NCD, with a total of 35% of women reporting being affected by ≥1 NCD (Figure 6). The proportion of reported NCDs varied depending on the outlet, with Patte d’Oie/Amitié 2, the most urban site, recording the highest proportion (45%), Thiès/Pout Diack the lowest levels (28.3%), and 31.7% in the periurban Toubab Dialaw/Bambilor. Women aged 46 to 49 y were the most affected, with 43.6% reporting ≥1 NCD, followed by the 35 to 45 (31.9%) and 25 to 34 (31.6%) age groups, with similar proportions. Younger women, aged 18 to 24 y, reported the lowest levels, with 27.8% declaring an NCD. Overall, the prevalence of NCDs increased with age, in line with medical research showing NCD risks increase with age.

In terms of type of NCDs, hypertension is the most common declared pathology, affecting 24.4% of respondents (Figure 6). It is followed by chronic respiratory diseases (7.8%), diabetes (3.3%), obesity (2.2%), and, lastly, cardiovascular diseases, which are the least frequent, accounting for only 0.6% of cases. These figures of self-reporting NCDs are far below those from the national statistics.

#### Consuming agroecological products to prevent diet-related NCDs

An analysis of women’s views reveals that those who consumed agroecological products for their health did so either as a preventive measure, aware of risks of diet-related diseases, or after having been diagnosed with an NCD. Some women reported using these products to prevent illness, whereas others used them to treat their illnesses and stabilize their state of health. Indeed, women who used agroecological products to prevent disease often had a family history of transmissible diseases such as diabetes. Therefore, they turned to these products to prevent these ailments, believing that avoiding ultraprocessed foods and foods containing pesticides and chemical fertilizer residues can help prevent the onset of these diseases.

*“Coming from a family affected by diabetes, my mother is diabetic herself. That's why I use this particular product a lot. Most processed foods do indeed pose health problems, which is why I prefer to turn to agroecological products in order to avoid this disease.” ESS_FD_C_Patte d'Oie*.

Among those who consumed agroecological products to treat their ailments or maintain their health, the majority were suffering from hypertension, constipation, or joint problems. These women felt that their health problems were mainly attributable to their diet. It is for this reason that they opted for agroecological products, or that someone close to them suggested that they give priority to this type of product.

#### BMI for women

**FIGURE 7:**
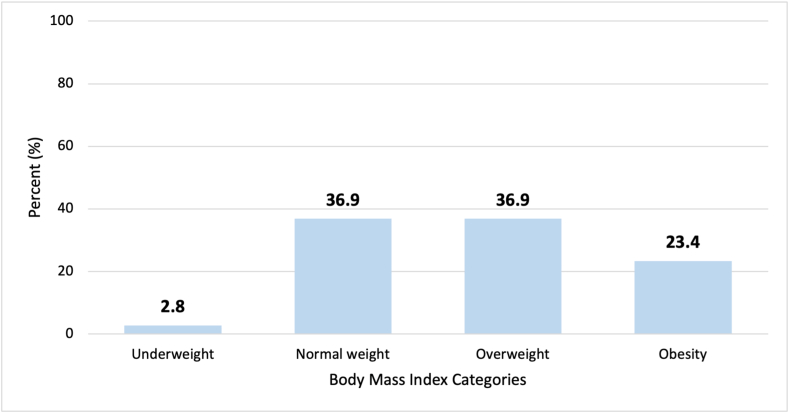
Categorization of female consumers by BMI (*n* = 180 respondents).

The results of BMI measurement show that figures of overweight and obesity were much higher than what was self-reported. Overall, there were 36.9% of women consumers with normal weight, and 36.9% who were overweight, whereas 23.4% were obese (Figure 7). There was a high prevalence of overweight and obesity, especially in Patte d'Oie/Amitié 2, which may be linked to urban eating habits, notably with the influence of a food environment marked by the presence of fast food and ultraprocessed foods. Conversely, Thiès/Pout Diack and Toubab Dialaw/Bambilor showed slightly different trends, with a higher proportion of people with normal weight.

### General eating habits and diets of women consumers

#### Frequency of food consumption

**FIGURE 8:**
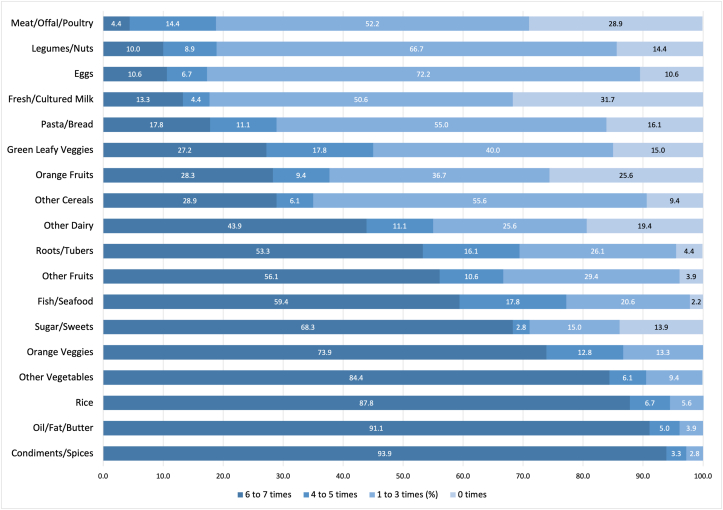
Percentage of women consuming each food group.

The frequency of consumption of different food groups (Figure 8) highlights a diversified diet dominated by cereals, fats, and vegetables, with significant consumption of fish, whereas animal protein sources such as meat and dairy products occupy a marginal place in women’s diets. Condiments and spices, oils and fats, and rice were the foods eaten most frequently over the last 7 d (6–7 times) by almost all women. Vegetables, particularly orange vegetables and other vegetables, also featured prominently in the diet: 6 to 7 times a week for the vast majority of women. Among animal products, consumption of fish and seafood was relatively high (6–7 d a week) for ∼60% of women, unlike meat, offal, poultry, and fresh or curdled milk, which were consumed much less frequently (1–3 times a week by half of women). Pulses, nuts, and eggs were also less common in women’s diets, consumed only 1 to 3 times a week by more than two-thirds of all women.

Table 4 shows the frequency of consumption of agroecological products according to different food categories. Condiments and spices are consumed most frequently on a daily basis. Green leaf, orange, and other vegetables are also eaten relatively frequently. Roots and tubers are a regular part of the diet. Orange fruit and other fruits were eaten daily by a lower proportion of women.

**TABLE 4:** Frequency of consumption of agroecological products (*n* = 180 respondents), in %.

Categories	Every day	A few times a week	Rarely	Never	Not concerned	Total
Roots, tubers	21.7	36.1	24.5	13.3	4.4	100.0
Orange vegetables	26.1	37.3	26.1	10.6	0.0	100.0
Green leafy vegetables	17.8	49.5	16.1	1.7	15.0	100.0
Other vegetables	27.8	34.5	28.3	9.4	0.0	100.0
Orange fruit	18.9	17.8	29.4	8.3	25.6	100.0
Other fruit	26.1	28.9	28.3	12.8	3.9	100.0
Condiments/spices	62.2	18.3	13.4	6.1	0.0	100.0

### Women’s motivations for consuming agroecological products

**FIGURE 9:**
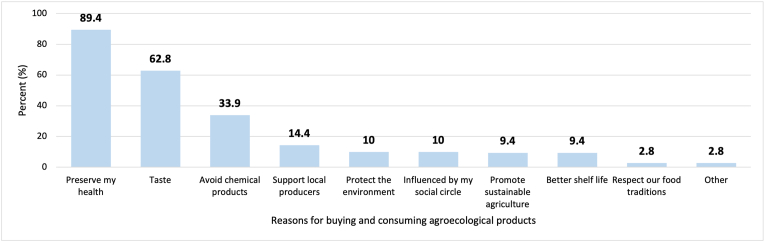
Main reasons for buying and consuming agroecological products (*n* = 180).

There were several reasons women gave for consuming agroecological products (Figure 9). Health was the dominant factor, with taste the second most important criterion, followed by food safety, particularly the avoidance of agrochemicals. Other reasons, although less frequent, were also mentioned: to support local producers, to protect the environment, and to encourage sustainable agriculture, or because of better food preservation. The influence of friends and family was mentioned by 10% of respondents, whereas 2.8% cited respect for food traditions or other reasons.

The women were also asked to identify their primary source of motivation from among the various options they had chosen. Health and taste stood out as the primary reasons, with 74% and 14.6% of respondents, respectively, placing them at the top of their list of motivations, whereas environmental and social aspects remained secondary in the decision-making process. A few consumers said that they turn to agroecological products to preserve the environment, because these do not use chemical products for their cultivation but rather natural substances.

### The potential for agroecological markets to support healthy diets for low-income urban households

The potential for agroecological markets to support healthier diets among low-income urban households is analyzed along the 6 dimensions of the analytical framework presented above. The analysis is informed by convergent results from both the market and the consumer components (quantitative and qualitative data).

### Availability

Both surveyed producers, vendors, and consumers raised the problem of low volumes of produce, with a limited land area for production, and a limited number of producers committed to agroecological production. A second challenge was the seasonal availability of the produce. Vendors in Thiès explained that during the high season (i.e., dry season), when production is abundant and prices are low on the conventional market, they do not have any difficulties obtaining requested volumes but may lack customers to sell 100% of the farmers’ produce on the organic market. In the low season, that is, the rainy season, production is reduced and prices increase sharply at the conventional market. The vendors face difficulty in supplying enough food because producers tend to sell to the more remunerative conventional market or to organic distribution networks in cities with higher purchasing power, such as Dakar or the tourist region of Mbour. The NGO Agrecol tried to organize a seasonal cultivation plan to stagger the production and thus avoid the disruption of certain products at the market. Nevertheless, the options remain very limited in the rainy season. These shortages can affect customer satisfaction, with some perceiving that this lower seasonal diversity was one of the reasons for reduced consumption, even if loyal customers tended to adapt, as one consumer from Bambilor noted:

“*Depending on the season, there are not certain products, but that’s how it is, you have to get used to it, it’s normal, we will not have tomatoes for a period, and we will not have carrots during wintering, and this, we must accept it. It is normal to say so and find other alternatives*.” ESS_MDS_Toubab Dialaw/Bambilor

Consumers buying agroecological products, though most of them (67.8%) reported no particular difficulties, indicated the limited availability of products as the most frequently mentioned problem among those who expressed difficulties (21.1%). Another notable difficulty was the distance to market (12.8%). Other constraints, such as lack of transport, incompatible opening hours, high travel costs, and lack of information on sales locations, remain very marginal, each cited by only 1.1% of respondents. Most women consumers surveyed considered that agroecological products are either “always available” (45.6%) or “fairly available” (46.1%) during the dry season. In contrast, the majority of respondents (76.7%) felt that agroecological products are “not readily available” during the rainy season. The types of agroecological products that are less available vary depending on the specialization of the market outlet; the garden in Patte d’Oie, for instance, specializes in condiments and spices (Table 5). Generally speaking, products that are frequently unavailable across all outlets include rice, pasta, and roots and tubers. Conversely, condiments/spices, green leafy vegetables, and other vegetables are considered more widely available, regardless of site.

**TABLE 5:** Agroecological food products least and most available by sales outlet (*n* = 180 respondents).

Agroecological market	Items requested but not available (%)		Most available items (%)	
Patte d'Oie/Amitié 2	Other vegetables	60.0	Condiments, spices	90.0
	Orange vegetables	46.7	Green leafy vegetables	66.7
	Orange fruits	36.7	Meat, offal, poultry	38.3
	Other fruits	28.3	Other vegetables	23.3
	Roots, tubers	25.0	Orange vegetables	8.3
Thiès/ Pout Diack	Orange vegetables	41.7	Other vegetables	76.7
	Rice	26.7	Green leafy vegetables	70.0
	Pasta (local)	26.7	Orange vegetables	66.7
	Meat, offal, poultry	26.7	Other fruits	56.7
	Fish/seafood	25.0	Condiments, spices	56.7
Toubab Dialaw/Bambilor	Other cereals	51.7	Orange vegetables	70.0
	Bean, pulses, nuts	36.7	Other vegetables	68.3
	Rice	30.0	Green leafy vegetables	60.0
	Pasta	26.7	Roots, tubers	55.0
	Roots, tubers	20.0	Other fruits	51.7

### Physical access

The location of sales outlets affects the physical accessibility to agroecological food products. In Patte d‘Oie, sales took place mainly at the production site in a Dakar neighborhood, along a busy road close to public transport stops, which makes it physically accessible to many people in the community or on their way to work. In Thiès, the location of the organic market was chosen by the NGO Agrecol and the women’s network, with the municipality providing land that was near the NGO offices. There was also a desire not to be directly in the central market to avoid any confusion between organic and conventional sellers. This location makes them less visible than the central market. In Toubab Dialaw, the women farmers sell in the local market, which makes their products accessible to the whole community, and during the monthly special events “Market Biodialaw.”

Another widespread practice in these agroecological marketing channels, which began during the COVID-19 outbreak, is home delivery via orders on a dedicated WhatsApp group or by phone. The latter is more informal, based on loyalty between producers and clients, and facilitated by money transfers. In both cases, they are more flexible than orders via the Internet and could therefore reach more clientele.

The question of the market’s location also raises a tension between making agroecological products physically accessible to low-income people (by selling in low-income neighborhoods) and farmer/vendor livelihoods. As 1 trader noted, selling in Dakar is more beneficial because she can sell higher quantities, especially nonindigenous vegetables, to people with more varied incomes, including higher incomes, and from different cultural backgrounds who eat a wider variety of vegetables, but this means that low-income Bambilor households miss out.

There was considerable variation in the distance that consumers had to travel to the agroecological markets. In urban Patte d'Oie/Amitié 2, most respondents (68.3%) considered the markets to be very or fairly close. In urban and rural Thiès/Pout Diack, only 35% of respondents considered the outlets to be close, with 50% considering the distance to be “average.” In contrast, in periurban Toubab Dialaw/Bambilor, most respondents (70%) rated the agroecological markets to be “quite far” or “very far.” Women used a variety of means of transportation to reach the various outlets, ranging from walking on foot to using personal cars, public transit, or taxis. Overall, regardless of the point of sale, most women (68.7%) walked to buy agroecological products. In qualitative interviews, nonconsumers of agroecological products indicated that accessibility was a major barrier for them to purchase agroecological foods.

### Affordability

Ensuring the availability of diverse food products for all is particularly important for the agroecological principles of *fairness* and *governance* processes, which respect people’s right to access healthy food products. Producers and consumers in this study both expressed support for these principles, but seasonal availability had a direct impact on prices with agroecological products. Conventional prices varied markedly between the dry season, when they dropped because of high production volumes, and the rainy season, when they rose because of lower production. Agroecological prices were more stable, because they were based more on production costs rather than the market dynamics. Because agroecological prices were more stable, they could be lower than conventional foods in the rainy season, as 1 vendor observed:

“*During the dry season, producers prefer to bring their produce here [to the organic market], because the products are cheaper on the conventional market. Currently [during the rainy season], our products are much cheaper. [...] But it's during the dry season that products are much more plentiful and cheaper on the conventional market. But our products are cheaper during this rainy period. The problem is product availability, but when we have the item at that time, our goods are less expensive”*. (Interview, Thiès, vendor)

In all cases, the question of setting prices was a complex one, with the agroecological principle of fairness in tension to support both decent livelihoods for small-scale family farmers and physical, social, and economic access to healthy food for all. During the rainy season, there was more competition between different agroecological markets because of the lower availability of the products. This competition meant that vendors from Dakar sometimes traveled to periurban and rural areas to purchase agroecological foods, as 1 NGO respondent noted:

*“So there are some who don't follow us, who say, OK, I've seen my carrot. So I can sell at a better price in Dakar. Yes, we have them too. We've had to deal with this problem. There's one producer, for example, when we give him the local market price, he says, no, no, no. People from Dakar come here to buy it for so many francs. And you want me to sell it to you for that?! So, I'd rather sell to them than to you”.* (Interview, Thiès, NGO)

All the sites were frequented by clients of different income levels. Several strategies were used by farmers and vendors to guarantee economic access to agroecological products to all, namely: *1*) limiting retail prices compared with those on conventional markets and *2*) decreasing the minimum purchase amount or adjusting prices. Some of the markets either limited the price or had a fixed, stable price to make the products affordable. In Thiès, farmers, vendors, and NGOs claimed to offer prices equal to, or slightly higher (50 or 100 CFA) than conventional prices, to make their products accessible to all. Each week, the NGO Agrecol Afrique provided producers, vendors, and consumers with recommended retail and wholesale prices for each product, based on the Market Information System, with a premium of 50 to 100 CFA per kilo. The pricing policy seemed to be widely followed by farmers and vendors. Despite these efforts and good intentions, some vendors expressed feeling aggrieved and trapped between producers seeking recognition for their quality work at more remunerative prices than conventional prices, and a “pricing policy” of the initiative seeking affordable retail prices.

In contrast, in Patte d’Oie, the producers said that their selling prices were certainly higher than on the conventional market, which they justified because of the quality (i.e., healthy and tasty) of their products. Furthermore, the minimum amount of produce sold is normally 500 CFA; however, some women agreed to sell only 200 CFA to those who could not afford 500 CFA. In a context where the low-income consumers buy their food daily (and in very small quantities), this practice is an interesting lever for improving access to these products for all.

According to Bambilor’s actors, the prices offered for agroecological products are equal to or higher than conventional prices. Profiles of customers are varied, but the price can make it difficult for the poorest ones to buy. The farm gate clients often buy a crate and then share out the goods. This practice is made possible by the good storage capacity of agroecological products, widely recognized by the interviewees.

Several interviewees (sellers or producers who do direct sales) suggested that prices vary according to customers’ perceived or stated budgets. This mechanism could be linked to a form of solidarity pricing (i.e., the wealthier paying a higher price so that the disadvantaged one can benefit from an affordable price), but it was neither institutionalized nor justified as such. [In Burkina Faso, an initiative experimented with this system of pricing (La Saisonnière).]

*“We have all sorts of customers... everyone buys according to their income. We used to sell at much higher prices, but to reach the poorest people, we've lowered the price and we also sell in small quantities. Now, when you come to the garden to buy, we can even sell you 100 francs worth of each vegetable.”* (Interview, Thiès, vendor)

In the context of the study, the idea that organic food (often used interchangeably with agroecological food) is for the rich is very widespread, so even if the products are made physically accessible and affordable, the most disadvantaged will not necessarily buy from these markets. This idea appears to be a major obstacle to increasing the consumption of agroecological produce among the poorest population. Despite these challenges, the 4 initiatives claimed their clients are from all income categories of consumers, although the proportion is unclear, as this respondent indicated:

*“We have different types of consumers. Honestly, in the market too, we have consumers who are really foreigners. Foreigners are really... westerners, who know the market well, who come and buy regularly. That too. There are some who are a bit more... educated like me, who went to school, to university, who know a bit, who come too, who buy. There are also some who are here for health reasons. They're really... They just want to be well-stocked, to eat healthily. There are also those who are not rich at all. They're less well off, but they believe in it and regularly, for example, there's... [Name of a particular client]. There's a guy who comes to every market, he's got 500 francs, he takes 200 francs worth of carrots and peppers and puts it in his bag. That's what he uses that week. And he does it every Saturday.”* (Interview, Thiès, NGO)

The consumer survey showed that agroecological products are considered highly affordable by most women consuming agroecological foods. The foods fit into the eating habits of 2 of 3 women (67.8%) without financial sacrifice, with 28.9% indicating that a financial effort was needed to obtain these foods. A small proportion (2.3%) found the products too expensive for their income. At the same time, most respondents (83.9%) stated that agroecological products are more expensive than conventional products, a perception particularly pronounced in Patte d'Oie/Amitié 2, where 90% of respondents found agroecological products to be more expensive. In Thiès, this proportion was also high (85%), whereas in Toubab Dialaw/Bambilor, it was slightly lower (76.7%), with a notable proportion of respondents (11.7%) believing that prices are cheaper. These results clearly indicate that most women, regardless of location, perceive agroecological products as generally more expensive during the rainy season, which could be a barrier to their widespread purchase.

### Acceptability

The agroecological markets provided a range of culturally relevant products, such as mint, spices, and locally grown grains such as rice and fonio. Although many of these products are not indigenous crops, they are still incorporated into local diets. One exception was the initiative in Bambilor, which emphasized the importance of culturally relevant foods.

Taste was mentioned as the second most important motivation (after health) to buy agroecological products and was mentioned far more frequently by women with limited education (28.6%) than by more educated women (11.2%). This finding suggests that agroecological markets meet the acceptability dimension of food environments and address important cultural values. In interviews, consumers also stated that the agroecological food products are much tastier than conventional products, which is why they prefer agroecological food.

*“I have this inexplicable feeling. I'm really happy when I eat agroecological products. I really feel like I'm eating. Sometimes my child says to me: Mommy, when I eat, I feel like I'm eating really well. I tell her it's because it's agroecological. I'm really active when I eat agroecological products because I know that what I'm eating tastes really good*.*” (*Interview, Thiès/Pout Diack, consumer)

In terms of quality and durability, women consumers indicated that agroecological products last longer than conventional products, do not have to be refrigerated, and can be stored in the open air.

*“Quality first, then sustainability. If you buy agroecological products and others containing chemical fertilisers and put them somewhere, the agroecological product will stand up much better than the conventional one. I've already made the comparison. If you happen to buy lettuce from agroecology and lettuce from conventional agriculture, the former can remain unchanged for 2 days, without rotting, unlike the conventional lettuce which, after a day, completely loses its aesthetic appeal. It's this comparison that allows me to say that agroecological products are better*.*” (*Interview, Thiès/Pout Diack*, consumer)*

Another less common reason given was to support local producers. Some claimed that by prioritizing the purchase of agroecological products, they can support local producers by sourcing their goods rather than importing them.

“*As I said, it's to support local producers and I favour local trade and community development. So, it's products from Toubab Dialaw next door, plus they're healthy and clean.”* (Interview, Toubab Dialaw/Bambilor, consumer)

### Market information

Trust was an important theme for producers, vendors, and consumers because there was very limited information on food production methods. Only 1 monthly market had signs; the other market sites had no visible signs to indicate sales of agroecological products. This lack of advertisement made the promotion of agroecology more complex, because consumers could not distinguish between agroecological and conventional markets. The initiatives only worked by establishing trust and a good reputation through interpersonal relationships and word of mouth. In the relationship between vendor and consumer, the vendors saw part of their role in communicating and justifying prices and product quality. They felt they were listened to, and customers agreed to buy, despite sometimes an initial hesitation due to the price. After that, it was the product experience that played a role: people tasted and came back. The place of trust and interknowledge as a catalyst for this trust can be illustrated by the sentence: “*They know the field and the people who sell the products. They know it's organic*” (Interview, Thiaroye, farmer).

Most consumers (88.3%) cited friends or family as their main source of information about the agroecological markets. Local events such as markets and fairs were a source for 8.9% of respondents, whereas social networks accounted for 6.7%. Less important sources were other sources (7.8%), NGOs (3.9%), the internet (2.2%), and advertising via posters or newspapers (0.6%). Most Patte d'Oie/Amitié 2 consumers said that they trusted the producers of agroecological products, which may be partially explained by their proximity to the garden. In this site, most vendors grew their own produce on their terraces. The proximity to sellers thus reinforced consumers’ confidence in consuming nonlabeled items that were nevertheless called agroecological products.

*“I trust the growers. They're all our moms, we live with them in the same neighbourhood, so if they say it's agroecological, I really believe it, I don't doubt it. In the whole locality, they're the only ones growing agroecological crops; the other horticulturalists on the other side use fertilisers and pesticides. But not the women who work at HLM Patte d'Oie.” (*Interview, Patte d'Oie/Amitié 2*,* consumer)

Nonconsumers of agroecological foods also attached great importance to trust in agroecological products and producers. According to some nonconsumers, their lack of trust in agroecological food producers led them to prefer conventional products. They perceived no difference in quality between agroecological and conventional products. Gaining and maintaining trust in agroecological methods thus emerged as a crucial aspect of food environments for low-income consumers in urban and periurban markets in this case study.

### Food quality and safety

Health concerns were emphasized as the main motivation for both consumers to buy agroecological products and farmers to use agroecological practices, and as a reason for vendors to sell agroecological food products. Three distinct health concerns are highlighted: food safety, diseases, and healthy diets.

#### Food safety

Avoiding chemicals (i.e., fertilizers and pesticides) for health reasons was one of the main arguments put forward by producers, sellers, and consumers. Reducing or eliminating the use of chemicals, in particular pesticides, was highlighted by promoters of initiatives and producers as a way to produce healthy food, protect the environment, and the health of consumers and farm workers. Some consumers also pointed out that their motivation for buying and consuming agroecological products is the fact that producers do not use chemical fertilizers or pesticides, which is why they prefer to buy and consume these products.

*“When a person eats, the whole organism is put to work. Each organ fulfils a well-defined role. However, a diet full of pesticides exhausts the organs, which must regulate and correct excesses. This is why many health problems are linked to our diet, such as kidney disease requiring dialysis, or cancer, largely caused by poor nutrition. Conversely, an agroecological diet offers better health and nutritional quality*.*”* (Interview, Toubab Dialaw/Bambilor, consumer)

Risk of biological contamination (e.g., bacteria) was not mentioned, even though this still can occur as products deteriorate or due to a lack of hygienic practices. The lack of mention of biological contamination might be due to the perceived good preservation capacity of agroecological products mentioned by many respondents. Finally, not using wastewater is promoted as a healthy practice in Patte d’Oie and Bambilor, unlike other production sites in the capital.

#### Diseases

Consuming agroecological or organic foods was viewed as a preventive action for health. Limiting risk of diet-related NCDs (diabetes, hypertension) was promoted during sales, and, according to the interviewees, was also a motivation for some consumers (on the advice of their doctor or relatives). NGOs supporting initiatives such as CICODEV have built their strategy to fight against these diseases on an agroecological approach. Other types of diseases that can be diet-related (e.g., cancer), and can be prevented or cured by healthy plants (e.g., insomnia, digestion, arthritis, vision) were also mentioned.

#### Healthy diets

Agroecology is seen by vendors and producers to promote healthy diets, either through producers consuming their own produce or through the sale of chemical-free, fresh, nutritious products to consumers. Selling a wide variety of vegetables and herbs is one way to support healthier diets and, for example, to use them in broths, such as for cooking fish, and replace industrial bouillon cubes. One NGO also emphasized the contribution of agroecology to sustainable dietary changes for children (e.g., deliver vegetables to canteens, not using “bouillon cube” and communicate this information, produce honey and partner with women vendors in front of the schools so that they can sell such honey bars to replace industrial foods, adapt packaging of nutritious local products such as milk), including diversified and seasonal diets. Adapting consumption to the local agroecological supply—and therefore taking seasonality into account—has been identified as a lever for action and a value to be promoted.

*“We communicate a lot with consumers, telling them: if the product isn't available, but listen, let's try to find another substitute, but don't go to the conventional market. So that too is a form of communication. And some people accept it. There's no carrot. They say, OK, I won't take a carrot, but I'll see what else I'll take apart from the carrot. For me, for example, that's how I do it: when there are no carrots at the market, I don't have any carrots. I don't eat carrots at home.”* (Interview, Thiès, NGO)

Among women with nonformal education, health was cited by 68.6% of respondents. Interestingly, they attach little importance to other factors, such as avoidance of chemicals (2.9%). Among women with formal education, health was also the main motivation (75.5%), but they also gave priority to other factors. They were more likely to mention the avoidance of chemicals (5.6%), the influence of those around them (1.4%), and better food preservation (1.4%). They were also more inclined to support local producers (4.2%), a reason that was absent among women with nonformal education. Qualitative data also revealed how women replaced certain processed or ultraprocessed products, such as bouillon and flavored rice, with agroecological foods. They asserted that these products helped them to avoid certain health problems.

*“People have to take a good look at the products they consume, and I'm convinced that when you use agroecological products, you won't run into any health problems, as long as they don't contain chemical fertilisers and are healthy. That's really why I prefer to buy agroecological products.”* (Interview, consumer, Thiès/Pout Diack)

Women consumers in the survey reported changing their eating patterns since the introduction of agroecological products into their consumption habits. More than half of the respondents (53.3%) reported eating more vegetables, and 43.3% reported an increase in their consumption of herbs and spices. A quarter of the participants reported consuming more fruit, whereas 14.4% stated that they paid more attention to the origin of their food. Most respondents (56.7%) reported that they now cooked more often with fresh produce, whereas 24.4% used fewer processed products. About 1 in 5 respondents (20.6%) said that they had experimented with new, healthier recipes. A total of 45.6% of women reported using less oil since consuming agroecological products, whereas 18.3% reported maintaining their consumption, 33.3% of respondents indicated that oil use depends on the type of meal being prepared, and 2.8% said that they did not pay attention to the amount of oil used.

Bouillon, or broth, is a high-salt ultraprocessed food addition widely used in cooking in Senegal, and 48.3% of women consumers reported using less broth after switching to agroecological foods, whereas 16.1% said that they still consumed the same amount, and 17.8% used even more. Only ∼12.8% of respondents stated that they had never used bouillon, and 5% adjusted their consumption depending on the meal type. Although most women report a change in their cooking habits, a significant proportion have observed no change (17.8%).

### Policy conditions

In the 4 agroecological initiatives studied, the key role played by local or national authorities and NGOs is noteworthy, and was valued by all interviewees: providing human resources to help the women farmers (Patte d’Oie), market sites (Thiès), or land to grow (Patte d’Oie). They were involved directly as partners of a project, or through encouraging the creation of a committee dedicated to food systems governance.

### Conceptual framework on impact pathways

**FIGURE 10:**
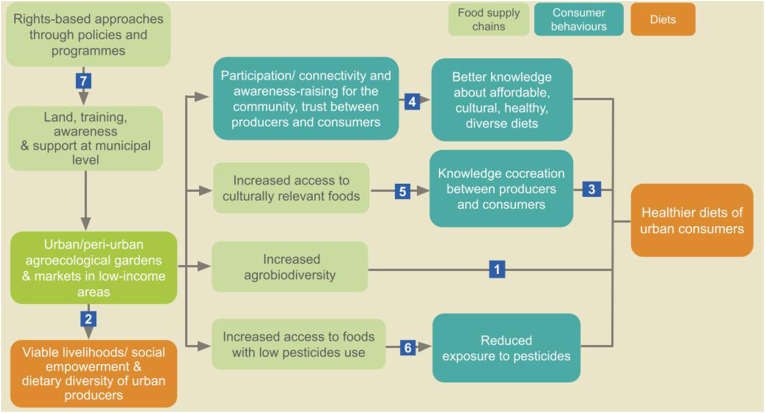
Conceptual framework for agroecology to improve nutrition of low-income urban consumers.

In this case study of Senegal, based on the literature review and our fieldwork in urban and periurban areas, we find ≥7 possible pathways for agroecology to support improved nutrition for low-income urban households (Figure 10). These pathways are as follows: *1*) agrobiodiversity, *2*) livelihoods/social empowerment, *3*) local knowledge systems, *4*) participation/connectivity, *5*) cultural foodways, *6*) reduced exposure to pesticides, and *7*) rights-based approaches. Although many of these pathways are the same as those for rural consumers, the participation/connectivity and reduced exposure to pesticides are different for urban consumers.

Agroecological principles can influence multiple pathways, and a given pathway can be affected by multiple principles, so the conceptual figure does not have direct linkages between each pathway and principle. Pathways are all within food environments, including food supply chain aspects, consumer behaviors, and diets. We will outline each pathway in turn.

*Pathway 1—agrobiodiversity:* consumers and urban producers themselves gain access to urban/periurban agroecological markets with a high diversity of species of vegetables/fruits and herbs, as well as varieties, including both indigenous and nonindigenous species or varieties. The intended benefits of supporting agroecological farming with a diversity of crops may only materialize if they are accompanied by addressing current dietary trends and preferences, in part because customers and farmers themselves may have limited knowledge of different varieties and be reluctant to try cooking unfamiliar foods, as shown in urban South Africa [49].

*Pathway 2—viable livelihoods/social empowerment*: farmers and vendors gain a viable income from their involvement in agroecological channels, and in turn may spend some of their income on healthy, diverse foods. In some of the agroecological markets, low-income women were working as farmers and vendors. These activities enabled women to gain money and to contribute to the needs of their families and the community, especially through mint, which is a highly remunerative crop. Notably, however, without additional interventions, unequal gender patterns of decision-making and control around income use may persist, and increased workloads might worsen nutritional status (Bezner Kerr et al. [10], 2019; [50]. This pathway might also require additional interventions to avoid the predominant shift in diets toward ultraprocessed foods high in fat, sugar, and salt.

*Pathway 3—knowledge cocreation:* horizontal and vertical sharing of knowledge on food and health are promoted, through farmer-to-farmer exchange or producer/vendor–consumer exchanges. In Thiès, these exchanges are organized by the NGO Agrecol Afrique, with peer visits and experience sharing. The initiative supported by CICODEV in Bambilor is similar: the CIGA has signed an agreement with the local youth center, which is providing 1000 m^2^ of land where 10 youth and 20 women will be trained in agroecological horticulture. In Patte d’Oie, this farmer-to-farmer exchange was driven by the FAO initially but has since become autonomous. Members of the community come and ask to be trained by women of Patte d’Oie, sometimes paid for by the city of Dakar, sometimes by the trainees themselves. Vertical sharing experience between producers/vendors and consumers includes nutrition and health benefits of agroecological products and culinary knowledge exchanges, especially regarding food preparation of underutilized species (e.g., spices, spinach).

*Pathway 4—participation/connectivity*: building greater participation and raising awareness about food systems issues—including malnutrition—for the whole community appears important for all the initiatives studied. In Bambilor, the CIGA adopted a wide-scale approach to sensitize the community (through schools and directly with someone visiting the community, a “visite à domicile”). In Thiès, they organized organic fairs 3 times a year and did radio broadcasts. In Thiaroye Gare, they built on the local *bajenu gox* (term in Wolof which means “neighborhood mentor”) who are a recognized and institutionalized relay between health centers and the communities. This social role of mentorship may contribute indirectly to consumer empowerment, especially linked to nutritional awareness. Direct and frequent interactions between producers and consumers in the agroecological markets establish trust and a common understanding of food issues. Vendor–consumer relationships are not limited to commercial relationships but also include a form of social bond—with long-term loyal clients—or sharing of culinary knowledge. The actors of these initiatives associate consumption of agroecological products with good health and communicate these values to their customers, thereby contributing to nutrition awareness.

*Pathway 5—culture and diets:* most interviewees emphasized the contribution of agroecological products to healthy, diversified, and seasonal diets rather than food that addresses cultural needs or values. Although the vegetables and herbs sold in the targeted markets are quite widely consumed, they include many nonindigenous vegetable species rarely used in traditional dishes, such as basil, beetroot, and cauliflower. The initiative by CIGA in Bambilor, however, shows how promoting traditional foods (e.g., local cereals like millet) and supporting Senegalese culture, such as avoiding bouillon cubes and promoting local herbs, can go hand in hand with agroecological products marketing.

*Pathway 6—reduced exposure to pesticides:* some NGOs and producers were focused on reduced exposure to pesticides as a key reason for their promotion of agroecological products. Reduced use of pesticides was expressed as a major motivation for the Ministry of Agriculture’s support of agroecology, as stated by the Department of Plant Protection representative (Interview, Dakar, policy maker). On the consumer side, one-third of consumers listed avoidance of pesticides and chemical fertilizers as a major motivation for choosing agroecological foods. In qualitative interviews, some respondents described specific health benefits and why reduced exposure to pesticides was a major motivation for their consumption of agroecological products.

We see this pathway through both a motivation for producers, programs, and consumers to buy agroecological foods such as fruits and vegetables, and via specific mechanisms, in that reduced exposure to pesticides may reduce the likelihood of type 2 diabetes and other diet-related diseases. There is considerable evidence of an association between exposure to organochloride or organophosphate pesticides and the development of type 2 diabetes [51,52], and emerging evidence that they can disrupt the function of adipose tissue to promote obesity and metabolic diseases such as type 2 diabetes [53]. There is also evidence that consuming organic diets is negatively associated with the prevalence of metabolic syndrome, a predictor of cardiovascular disease [54].

*Pathway 7—rights-based approaches:* the idea of offering healthy food to all is widely integrated in the discourse of development actors, including, in some cases, rights-based approaches. (Food sovereignty and right to food are part of the right-based approaches connected to agroecology. In an urban context, we can also include the right of cities which includes several rights that city residents require in order to enjoy adequate living standard. Ensuring the right to the city is guaranteeing urban spaces are inclusive, participatory and designed to meet the need of all the residents.) In Bambilor, CIGA takes this rights-based approach, with the aim to “democratize food” so that “*it’s no longer just a matter for the rich. Let the poor child know that he or she can eat healthy, and at the same time local food’*” (Interview, Bambilor, NGO staff). Part of a rights-based approach includes addressing policy conditions such as *access to land*, which was mentioned as a key lever to scale out agroecology, and an area of concern in the urban and periurban context. In Thiès, the women vendors were previously organic farmers in the city; they felt constrained by the lack of land, while they had the knowledge and skills to grow agroecological vegetables. They expressed their wish to access land to grow, or at least to train young people in agroecological methods. The space in Patte d’Oie was relatively limited and may be due to low land availability in an urban context. In Bambilor, which is a periurban location more distant from central Dakar, land availability was better, and they were able to obtain significant land for production. In Toubab Dialaw, access to land given by the “Ferme des 4 chemins” to the group of women farmers was key to their involvement in agroecological farming, in addition to the training received.

## Discussion

This case study has analyzed the functioning of agroecological markets in low-income urban and periurban areas, as well as consumption behaviors and diets of women purchasing in these markets to ultimately identify pathways by which agroecology could improve urban nutrition. Our study found that health, taste, and food safety were the main motivations for urban consumers to eat agroecological foods. This finding is well aligned with other studies identifying personal health problems, food quality (e.g., taste, freshness), and food safety as key motivations of urban consumers involved in alternative food systems such as agroecological markets [14,[55], [56], [57]]. The fact that some consumers were motivated by food safety in choosing to consume agroecological products is also in line with previous studies that found that agroecology means a “natural agriculture without chemicals” for most surveyed consumers in Dakar (64%) [58]. It must be noted that organic or agroecological practices are not automatically a guarantee of food safety, because biopesticides can also pose a risk at high doses [59].

Consumers of agroecological products are far from all being in good health; indeed, a substantial share of them (35%) reported being diagnosed with ≥1 NCD. In fact, this personal health experience, or knowing relatives and friends affected by NCDs, is precisely what has motivated many of them to decide to consume agroecological foods. Consumers of agroecological foods had diversified diets, and most of them reported having changed their consumption practices toward healthier diets, with more consumption of fruits and vegetables and less ultraprocessed foods, because they began going to agroecological markets. Other studies have documented changes in people’s diets after agroecological interventions, although notably, in these studies, the changes observed were in farm household diets [8,10,11].

There are several barriers to agroecology supporting urban low-income people’s improved nutrition. Availability and access were, in part, conditioned by seasonality in this study. Seasonal shortages of agroecological foods during the rainy season are in part due to increased pest and disease pressure with higher temperatures [60], but are likely also due to a combination of political, technical, economic, and epistemological factors that reinforce the primacy of chemical pesticides in horticulture in Senegal [61]. A 2019 report concluded that 67% of the vegetables sampled in the wholesale market of Thiaroye and 63% in the market of Notto were contaminated, with Codex Alimentarius maximum residue limits exceeded for ≥1 pesticide [62]. Several agroecological methods to control insects and diseases in horticulture in Senegal hold promise but are not widely used, including the promotion of natural enemies [63], traps [24], and pesticidal plant sprays [64].

Although agroecological production is challenging in the rainy season in this context, it is abundant and diverse in the dry season. Farmers are struggling to sell their produce as organic in dedicated markets, because high transport costs in cities may offset the small price advantages compared with conventional markets. In one of these markets, despite the NGO’s efforts to find a fair balance and satisfy both producers and vendors, the system remains fragile, with producers sometimes practicing side-selling (because of better prices in other outlets) and vendors facing supply and sales challenges, at times, increasing prices. This tension—related to the agroecological principle of fairness—of reconciling producer and vendor interests with a commitment to supplying the collective initiative to ensure a steady supply of local, affordable agroecological products is evident. The tension is also found in the choice of products, between the most remunerative crops (e.g., mint or vegetables with high retail price, such as green beans) compared with the most nutritious and culturally adequate. This question of affordability is critical because fruits and vegetables account for half of the cost of a healthy diet (data from 2017) [65]. These challenges are consistent with recent studies of territorial markets, which are typical of short food supply chains promoting family farming, market inclusivity for small-scale entrepreneurs and producers, and a direct relationship between consumers and producers [66,67].

Even though prices of agroecological products are not systematically higher than those of conventional products—it depends on the season—and low-income women consumers report buying agroecological products is not a sacrifice, the perception that agroecological products are “for the rich” is persistent. This finding suggests that barriers to increased consumption of agroecological products are not only the result of bottlenecks inherent to the current agroecological initiatives, such as seasonal availability, but also relate to the broader context and social representations of agroecology. A recent study of narratives about agroecology in Africa echoes the challenge of opposing persistent, if unsubstantiated, claims about agroecology [68]. Similarly, it seems difficult to develop agroecological markets for healthier diets without substantial actions on the broader food environment and on social norms regarding easy-to-prepare but nutrient-poor ultraprocessed foods.

Notably, in all the studied agroecological markets, an intermediary actor (e.g., NGO, municipal government) has played a key role in the emergence and sustainability of the initiatives, allocating land, training, and support, often with technical and financial partners. This finding is in line with previous studies that highlighted the key role of intermediaries such as local authorities in setting up a physical market space where agroecological products, as well as information and values, can be exchanged [38]. In Brazil, for example, the government contracted agroecological farmers to grow food for a public procurement program that supported low-income households and the national school feeding program [69]. Other scholars have been critical of the more top-down approach to governance that some in the NGO sector have taken to agroecology, likening it to a neocolonial approach [27]. Other studies have highlighted the need to include agroecology-oriented producers in municipal decision-making structures and governance [70]. The nearby example of Ouagadougou, Burkina Faso, setting aside 2000 hectares of urban land as a “green belt” around the city for agroecological food production and to reduce urban heat impacts (see: https://www.theguardian.com/environment/2025/feb/06/we-water-rest-water-the-green-belt-of-vegetable-plots-cooling-a-city), thereby serving as both a food security strategy and a climate change adaptation, is an example of governance strategies that could support agroecology within cities.

This study has identified 7 different pathways by which agroecological approaches could support improved nutrition for low-income households. Although some of the impact pathways align with those identified for rural households [10], we have also identified some new possibilities in urban contexts. Our analysis of agroecological markets highlighted the importance of trust, connectivity, and knowledge sharing—about nutrition and health issues in particular—between producers, vendors, and consumers. Although these characteristics may exist in conventional open-air markets, they are particularly strong in agroecological markets, making the experience of actors in these markets different from other markets. To maintain this specific experience while scaling up the marketing of agroecological products in low-income areas suggests that the multiplication of—even small—agroecological points of sales in these areas is a particularly relevant strategy vis-à-vis the strategy of increasing agroecological products in supermarkets.

The commitment of the leaders of agroecological market initiatives toward the values and social principles of agroecology, such as fairness and right-based approaches, reinforces the specificities of agroecological markets compared with conventional ones. Their effort in setting fair price mechanisms for both producers and consumers also shows that providing quality foods is not necessarily synonymous with quality premium and higher prices.

The impact pathways can materialize and deliver their full potential only if certain conditions are met or additional interventions are implemented, such as those mentioned in the description of pathways 1 and 2. On the basis of these findings, we developed recommendations to mobilize agroecology to address urban nutrition for low-income households (Table 6).

**TABLE 6:** Recommendations, possible actions, and rationale to mobilize agroecology for urban nutrition.

Recommendation	Example of action	Rationale
1. Support agroecological producers in an urban and periurban context	Increase access to land in urban and periurban low-income areas to increase agroecological production and access.	Urbanization threatens farmland in urban and periurban areas, which increases logistical problems, transport costs, and distance between producers and consumers.
	Farmer training, experimentation, and exchanges ensure seasonal availability of agroecological food products.	Addresses availability and affordability, especially for products subject to strong price variations.
2. Supporting short agroecological territorial markets/food chains.	Invest in small-scale and operational infrastructures (cold room, storage facility, for example).	Allows for a more regular supply of agroecological foods at market outlets.
	Invest in multiple small-scale kiosks or mobile markets in low-income neighborhoods.	Farmers and/or vendors could better reach vulnerable households.
	Support public procurement mechanisms of agroecological foods, for example, school foods, hospitals, community health clinic gardens, and/or public restaurants or canteens.	Increases consumer access to agroecological foods and provides market for producers.
3. Support transversal collective action (producers, vendors, consumers) to build a fair and long-term price-setting mechanism.	Price-setting mechanisms could include an agreement on a price band (floor and ceiling) for the year or the establishment of a solidarity price.	Addresses equity and fairness questions for both consumers and producers in agroecological urban markets.
4. Strengthening national and urban food governance and policy.	Development of a National action plan or Food Policy Council at the municipal level on agroecology that links health, agriculture, climate, and biodiversity policies and programs.	Longer-term redesign of food governance to make it more responsive to low-income consumers and agroecological producers.
5. Raising public awareness of agroecology links to health, culture, and livelihoods.	Promote awareness of agroecology and links to health, nutrition, and other agroecological values, such as justice, culture, and local livelihoods, through large-scale food campaigns in the public media, health centers, schools, urban community kitchens, cultural events, and other venues.	In some contexts, consumers cannot afford healthy diets, so raising awareness on nutrition would not be that helpful, but in countries like Senegal, where the cost of a healthy diet has been shown to be lower than the current average food expenditures, such action could have an effect.
6. Supporting participatory research on agroecology	Support more participatory research studies on agroecology impacts on urban nutrition and on diet-related diseases in different contexts.	There is a gap in the literature on agroecological approaches in urban settings and on the potential for agroecology to address chronic, diet-related diseases. Participatory research approaches allow for knowledge cocreation with marginalized groups.

In conclusion, this study aimed to examine potential impact pathways between agroecology and nutrition in an urban context for multiple forms of malnutrition, with a focus on low-income urban women. We chose Senegal as a case study, both because of the high rate of malnutrition and an active agroecological social movement, which provided potential avenues to address malnutrition. It must be noted that these findings are based on 1 case study in the Dakar region of Senegal and will not apply to every context.

We found that the marketing channel characteristics echo the connectivity principle of agroecology, where proximity and trust between producers and consumers are ensured through short distribution networks and re-embedding of food systems into local economies. Indeed, direct sales from producers to their neighbors seem to work well, thanks to relational and physical proximity.

On the other hand, the different markets also raise some of the tensions within the agroecological approach related to the principle of fairness, which seeks to provide decent livelihoods to producers while also providing affordable, healthy agroecological products. Agroecological initiatives need to strive to have grassroots participatory governance, including with low-income consumers, to ensure that they meet these divergent needs.

On the basis of the consumer survey, women consumers of agroecological products had higher than average levels of education compared with national rates, and the majority were overweight or obese, at a much higher prevalence than Dakar’s average. One-third of them reported having a diet-related disease such as diabetes or cardiovascular disease. The main motivations for consumers to purchase agroecological food products were health reasons, taste, and avoidance of pesticides and/or chemical fertilizers. Consumers noted several barriers to eating more agroecological foods, including the availability of markets, the distance to markets, and the seasonal availability of diverse food products. Consumers’ trust was based on interpersonal interactions with producers and sellers.

On the basis of this exploratory study, we identified 7 possible pathways to connect agroecology to nutrition. These pathways are as follows: *1*) agrobiodiversity, *2*) livelihoods/social empowerment, *3*) knowledge cocreation, *4*) participation/connectivity, *5*) culture and diets, *6*) reduced exposure to pesticides, and *7*) rights-based approaches. Although agroecology has the potential to address urban malnutrition for low-income households, there are multiple barriers and gaps that would need to be addressed. We identified 6 areas of action for such pathways to be enacted, namely supporting urban agroecological producers, expanding agroecological territorial markets, supporting transversal collective action for fairer prices, strengthening urban food governance, increasing public awareness, and carrying out further research on urban agroecology. Harnessing agroecology to address urban nutrition has strong potential to support healthy and diverse diets, if both production and demand sides of food systems are considered, as well as a broader policy vision that goes beyond food systems, particularly in supporting the livelihoods and incomes of vulnerable populations in urban, periurban, and rural settings.

## Author contributions

The authors’ responsibilities were as follows – RBK: designed, participated in qualitative data collection, led analysis and writing of the manuscript; MS: designed survey, led data collection of survey and focus groups, contributed to analysis and writing of the manuscript; AA: designed, participated in qualitative data collection, contributed to analysis and writing of the manuscript; NS: designed and led market survey, contributed to analysis and writing of manuscript; and all authors: read and approved the final version of the manuscript.

## Data availability

Data described in the manuscript, code book, and analytic code will be made available on request pending approval.

## Funding

The study was supported by the Nutrition Research Facility (NRF) within the Knowledge and Research for Nutrition Project funded by the European Union (2020–2026).

## Declaration of generative AI and AI-assisted technologies in the writing process

The author(s) declare that no generative AI or AI-assisted technologies were used in the writing of this manuscript.

## Conflict of interest

The authors have no conflicts of interest to declare.
